# Considerations for Training and Workforce Development to Enhance Rural and Remote Ophthalmology Practise in Australia: A Scoping Review

**DOI:** 10.3390/ijerph19148593

**Published:** 2022-07-14

**Authors:** Kehinde Obamiro, Belinda Jessup, Penny Allen, Victoria Baker-Smith, Santosh Khanal, Tony Barnett

**Affiliations:** 1 Centre for Rural Health, Newnham Campus, University of Tasmania, Launceston, TAS 7250, Australia; belinda.jessup@utas.edu.au (B.J.); tony.barnett@utas.edu.au (T.B.); 2 Rural Clinical School, Hospitals’ Campus, University of Tasmania, Brickport Road, Burnie, TAS 7320, Australia; penny.allen@utas.edu.au; 3 The Royal Australian and New Zealand College of Ophthalmologists, 94-98 Chalmers Street, Surry Hills, NSW 2010, Australia; victoriabakersmith@ranzco.edu (V.B.-S.); skhanal@ranzco.edu (S.K.)

**Keywords:** ophthalmologists, recruitment, retention, workforce

## Abstract

Australia has one of the lowest per capita numbers of ophthalmologists among OECD countries, and they predominantly practise in metropolitan centres of the country. Increasing the size and distribution of the ophthalmology workforce is of critical importance. The objective of this review was to investigate the context of rural ophthalmology training and practise in Australia and how they relate to future ophthalmology workforce development. This scoping review was informed by Arksey and O’Malley’s framework and the methodology described by Coloqhuon et al. The search yielded 428 articles, of which 261 were screened for eligibility. Following the screening, a total of 75 articles were included in the study. Themes identified relating to rural ophthalmology training and practise included: Indigenous eye health; access and utilisation of ophthalmology-related services; service delivery models for ophthalmic care; ophthalmology workforce demographics; and ophthalmology workforce education and training for rural and remote practise. With an anticipated undersupply and maldistribution of ophthalmologists in the coming decade, efforts to improve training must focus on how to build a sizeable, fit-for-purpose workforce to address eye health needs across Australia. More research focusing on ophthalmology workforce distribution is needed to help identify evidence-based solutions for workforce maldistribution. Several strategies to better prepare the future ophthalmology workforce for rural practise were identified, including incorporating telehealth into ophthalmology training settings; collaborating with other health workers, especially optometrists and specialist nurses in eyecare delivery; and exposing trainees to more patients of Indigenous background.

## 1. Introduction

Although Australia’s ophthalmology workforce comprises well over 1000 ophthalmologists, collectively, Australia has one of the lowest per capita numbers of ophthalmologists among OECD countries [1]. This situation is of concern given that future estimates suggest that ophthalmology is one of the medical specialties predicted to experience a critical shortage within the next 10 years [2]. It has been estimated that even with a yearly growth of 3% in the number of qualified ophthalmologists, there will still be an undersupply of up to 68 ophthalmologists by 2030 [3]. Another estimate has suggested a larger shortfall of 162 ophthalmologists by 2025 [4], highlighting inadequate funded training opportunities for ophthalmologists in the public sector, an imminent shortage of paediatric ophthalmologists and a potential dependence on international medical graduates to bolster the Australian ophthalmic workforce [4].

Although Australia collectively needs more practising ophthalmologists, the situation is even more critical in regional, rural and remote areas of the country where few medical specialists are located [3]. In 2018, Australia’s Future Health Workforce–Ophthalmology reported that 84% of ophthalmologists are in metropolitan areas, with the remainder practising in regional, rural and remote areas [3]. This implies that communities outside of metropolitan areas may have delayed or reduced access to ophthalmic care. Despite previous efforts and policies to improve eye health in non-metropolitan locations, people living in rural and remote locations are still disproportionally affected by eye diseases [5]. Therefore, there is a real need to develop a fit-for-purpose ophthalmic workforce that is both cognisant of rural eye health needs and capable of delivering care to those most in need.

One Commonwealth Government Department of Health initiative designed to support an increase in the size and distribution of the ophthalmology workforce in non-metropolitan areas is the Specialist Training Program (STP). In 2021, the STP funded 15 ophthalmology training posts that provide specialist vocational training in a range of private and/or rural settings across Australia [6]. The STP initiative broadly aims to enhance the capacity of the healthcare sector, providing access to specialist medical care to underserved communities, extending specialist training into healthcare settings experiencing workforce shortages and contributing to improving medical workforce distribution by encouraging medical specialists to return to areas of workforce need after qualification [7]. The program was established in 2010 by consolidating seven existing programs into a single program [7]. In 2015, the program was reviewed, with a number of major reforms implemented, including providing colleges with greater flexibility to manage the program to ensure it is responsive to emerging training needs [7].

Given the important role the STP may play in improving ophthalmic care in underserved areas, it is important to understand both the nature of ophthalmology training and ophthalmology service provision in rural and remote areas to ensure the development of a fit-for-purpose ophthalmology workforce that is skilled and able to meet the demand for services in this context. The aim of this review was therefore to investigate the context of rural ophthalmology training and practise in Australia and how they relate to future ophthalmology workforce development. A scoping review was conducted to provide an overview of a large and diverse body of literature that pertains to rural and remote ophthalmology practise in Australia.

## 2. Method

In order to provide a broad overview of the topic, a scoping review was conducted in accordance with a methodology described by Arksey et al. and Coloqhuon et al. [8,9,10]. Scoping reviews have been defined as a form of knowledge synthesis that incorporates a range of study designs to comprehensively summarize and synthesize evidence with the aim of informing practice, programs and policy and providing direction to future research priorities [9]. This review was conducted in collaboration with the Royal Australian and New Zealand College of Ophthalmologists (RANZCO) who are committed to the development of rural training to address the maldistribution of the ophthalmology workforce, improve eye health services in underserved locations and increase the number of Indigenous medical practitioners who become ophthalmologists [11].

### 2.1. Search Strategy

Electronic searches of PubMed, Embase, CINAHL, Google Scholar and Scopus databases were conducted to investigate the context of rural ophthalmology training and practise in Australia and how they relate to workforce development. The search terms were: (Austra * or New Zealand) and (rural or remote) and (recruit * or retain * or retention * or training) and (ophthal *). The scoping review initially focused on academic literature, with eligible reports included from the grey literature following a reference check of included papers.

### 2.2. Eligibility Criteria

Table 1 shows in detail the inclusion and exclusion criteria for the review. Included articles had to focus on rural ophthalmology practise or the training of ophthalmologists in rural and remote Australia. To focus on contemporary issues, articles were limited to the period from January 2005 to June 2020. The search was updated prior to finalisation of the review results in February 2022 to identify newly published articles.

### 2.3. Study Selection

**Figure 1:**
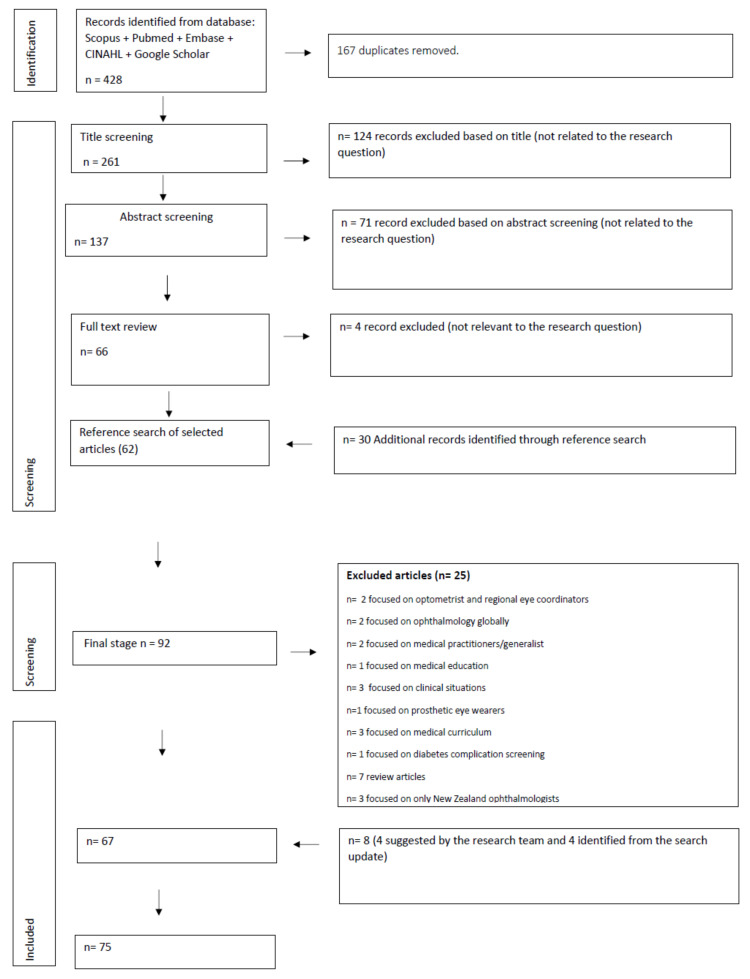
Flow chart of selection process.

On the completion of the database search, all citations were exported into EndNote X7 (Thomson Reuters, New York, NY, USA) reference management software, and duplicates were removed (Figure 1). In the first stage, two authors independently screened titles for eligibility using the inclusion/exclusion criteria (KO, BJ). Articles for which either reviewer agreed met eligibility were retained. In the second stage, the abstracts of included articles were again screened independently by four reviewers (KO, BJ, PA, TB). Articles for which either reviewer deemed the abstract to meet inclusion criteria were included. In the third stage, the full text of all selected articles was retrieved for independent screening by two members of the research team to determine relevance to the study question (KO, BJ). Articles were included if there was consensus by both researchers that it was relevant to the study objective. Disputed articles where researchers disagreed on relevance were referred to the broader research team, with consensus achieved through discussion (KO, BJ, PA, SK).

In the final stage, the reference lists of selected articles were searched for additional papers that may meet eligibility criteria and could be included. Additional papers found through this search were again subjected to an independent screening process, with two members of the research team determining their eligibility (KO, BJ). Any instances of disagreement were resolved through consensus discussion with the broader research team (KO, BJ, PA, TB).

### 2.4. Data Extraction Process

Relevant data from each article were extracted using an extraction template. The extraction sheet was designed for comparison and then to enable the synthesis of information across articles. After this, major themes were identified by grouping articles with a similar research focus together [12].

## 3. Results

A total of 428 articles were retrieved from the literature search (Figure 1). After removing duplicates, 261 articles remained. Further, 124 records were excluded from title screening, 71 from abstract screening and 4 from full-text screening. On completion of the reference check of eligible articles, 30 additional records were identified, of which 21 were excluded because they were not related to the research question (Figure 1). Following the methodology for scoping reviews, four additional articles that met the inclusion criteria were included based on the recommendation of members of the research team. This resulted in a total of 75 articles in this review. The articles included were 4 editorials, 2 policy papers, 2 reports from the grey literature and 67 original research articles. To identify major themes, data were coded, followed by the development of descriptive themes [12]. The major descriptive themes generated from the codes included: Indigenous eye health; access and utilisation of ophthalmology-related services in rural and remote areas; service delivery models for ophthalmic care in rural and remote areas; ophthalmology workforce demographics; and ophthalmology workforce education and training for rural and remote practise.

### 3.1. Indigenous Eye Health

The review identified 42 articles that reported on the incidence and prevalence of various eye diseases amongst the Indigenous population, including keratitis, visual impairment, vision loss, cataract, glaucoma, choroidal nevi, eye trauma, trachoma and diabetic retinopathy [13,14,15,16,17,18,19,20,21,22,23,24,25,26,27,28,29,30,31,32,33,34,35,36,37,38,39,40,41,42,43,44,45,46,47,48,49,50,51,52,53,54] (Table 2). Irrespective of the study design, these studies collectively demonstrated that the prevalence of vision impairment and eye diseases in Aboriginal and Torres Strait Islanders (respectfully referred to as Indigenous Australians) is consistently higher compared to non-Indigenous Australians. For example, a study by Keel et al. investigated the prevalence rate of near vision impairment using a sample of 3098 non-Indigenous Australians and 1738 Indigenous Australians [13]. A higher prevalence of near vision impairment was reported among Indigenous Australians at 34.7% versus 21.6% in non-Indigenous Australians. In a study investigating the prevalence of blindness and vision loss by Vos et al., similar findings were reported [14]. In this study, 1694 Indigenous children and 1614 Indigenous adults from 30 communities were included, with the prevalence of blindness and vision loss amongst adult Indigenous Australians found to be higher compared with estimates for the total Australian population. Further, the number of years lived with a disability was seven times higher among Indigenous Australians compared to the total Australian population [14]. A National Eye Survey by Foreman et al. was conducted to determine the prevalence and cause of vision loss in the Australian population [15]. The study included 1738 Indigenous Australians and 3098 non-Indigenous Australians. The prevalence of vision loss in non-Indigenous Australians was estimated to be 6.6%, while the prevalence was 11.2% among Indigenous Australians after adjustment for age and gender. Moreover, the results suggest that there are risk factors common to all Australians and those specific to Indigenous Australians. Advancing age and not having had an eye examination within the past year were identified as common risk factors, while additional risk factors among Indigenous Australians included remoteness, gender and diabetes. A study that investigated the prevalence of visually significant cataracts [16] reported the prevalence of visually significant cataract was higher in Indigenous Australians at 4.3% compared to 2.7% in non-Indigenous Australians.

Brazionis et al. found that 47% of Indigenous Australians living in remote areas had diabetic retinopathy, and 16.2% had sight-threatening diabetic retinopathy [17]. Lander et al. investigated the prevalence of pseudo-exfoliation syndrome among 1884 Indigenous Australians aged 20 years and above [18]. Pseudo-exfoliation syndrome was present in one or both eyes of 5% of individuals, and the prevalence was observed to increase with age. There are also reports that have reported a higher death rate in Indigenous people with eye conditions compared to the general Indigenous population. Ng et al., in a prospective study, assessed the association between visual impairment and 10-year mortality risk in a sample of 1347 Indigenous Australians from 30 remote communities [19]. The study showed that all-cause mortality was 29.3% at 10 years. After stratifying into those without visual impairment and those with visual impairment, all-cause mortality was more than two times higher in those with visual impairment. This result remained the same even after further adjustment for gender, age and medical conditions such as hypertension and diabetes.

### 3.2. Access and Utilisation of Ophthalmology-Related Services in Rural and Remote Areas

Twelve studies were identified that described issues relating to the access and utilisation of ophthalmology services in rural and remote areas [55,56,57,58,59,60,61,62,63,64,65,66] (Table 3). In terms of access, Foreman et al. compared the participation rate in routine eye examinations, finding that a lower proportion of Indigenous Australians (67%) compared to non-Indigenous Australians (82.5%) underwent an eye examination within the previous two years [55]. Factors associated with less recent examination included male gender and living in outer regional and very remote areas. A study by Boudville et al. investigated barriers that limit access to cataract surgery for Indigenous Australians using focus group discussions involving 530 participants [56]. The study identified cost, long waiting, times, complex processes involved in treatment and absence of surgical capacity as barriers to accessing treatment. A study by Arnold et al. similarly described how a lack of local service options impacted service access [57]. In their survey of Indigenous Australians residing in 30 remote communities, around 14% with vision problems did not seek help because eye services were not available in their area. A further 10% did not seek help due to the absence of suitable transportation or distance constraints. Turner et al. confirmed that service availability was an important factor in ophthalmic care utilisation, reporting that the availability of full-time equivalent ophthalmic services working in an Indigenous Medical Service led to a decrease in the prevalence of low vision and an increase in the coverage rate for distance refractive correction [58].

### 3.3. Service Delivery Models for Ophthalmic Care in Rural and Remote Areas

Several service delivery models were identified from the literature, including outreach services [67,68,69,70,71], collaboration with other healthcare professionals, including optometrists and nurses [72,73], integration of eye care with other locally available services [74] and the use of teleophthalmology [75,76,77,78,79,80,81] (Table 4). Research has identified that ophthalmologists are one of the most willing medical specialties to participate in service outreach to rural and remote communities [67]. O’Sullivan et al. conducted an Australia-wide study to determine the proportion of specialist doctors who participate in rural outreach [67]. Of 4596 specialist doctors recruited, 909 were involved in outreach programs, out of which 16% (149) were involved in remote outreach. Ophthalmologists were among the top five specialties involved in outreach services. Fu et al. reported on the impact of the Lions Outback vision mobile ophthalmology service van (LOVV) [68]. Sixteen regional towns were visited two or more times per year with total distance travel of about 25,000 km. Between 2015 and 2016, the number of outreach ophthalmology consultations was observed to increase by over 20%, and this enabled community members to benefit from many specialists’ ophthalmological instruments and expertise not previously available in any of the locations [68]. An article by Turner et al. described funding models used in outreach ophthalmology services and compared the impact on remuneration for clinical activities and cost-effectiveness [69]. The result showed that there is a greater than three-fold increase in costs per patient when comparing the most expensive and least expensive services. The study showed that funding approaches used can impact the effectiveness and sustainability of an outreach program. An ideal model should ensure the minimal cost to the end-user and an adequate remuneration rate for personnel providing eye services.

Other studies have explored the integration of ophthalmic care with other eye care providers in local communities. For example, a retrospective study by Glasson et al. suggests that an innovative model of diabetic retinopathy screening greatly improved accessibility in remote communities, thereby reducing preventable blindness [70]. The model provided a holistic, locally appropriate diabetes service and utilised existing infrastructure and health workforce efficiently. The model was found to be acceptable to both patients and health professionals [71]. A clinical audit of 300 patients by Slight et al. investigated if a nurse specialist could reduce waiting list numbers and waiting length for the first specialist assessment for glaucoma [72]. The introduction of the glaucoma clinical nurse specialist led to a significant reduction in waiting lists of patients, both those who fall within the nurse specialist’s area of practice and for more complex patients as appointments in consultant clinics freed up. A study by O’Connor et al. reported on the perspectives of optometrists, ophthalmologists and patients on a model of shared care for patients with chronic eye diseases [73]. Optometrists were not only able to meet ophthalmologists’ expectations but exceeded them by appropriately identifying and referring patients with previously undetected conditions. Patients mentioned savings in travel time as a benefit and were satisfied with the quality of care they received [73]. Optometrists, ophthalmologists and patients indicated a general acceptance of the shared care model [73]. Turner et al. described models of service integration between optometrists and ophthalmologists when conducting outreach eye services using nine outreach ophthalmology services [74]. Good service integration was observed to increase surgical caseload for ophthalmologists, reduce wait times and increase clinical services without impacting cost [74].

This review also identified seven articles related to teleophthalmology and showed teleophthalmology to be cost-effective, useful in managing a wide range of eye conditions and for training and mentoring, and beneficial across the continuum of patient care. Kumar et al. reported on the utility of an internet-based service for eye care in a remote Australian setting [75]. Among 118 remote participants in teleophthalmology consultations, the average consultation time was 30 min, with 86% requiring primary care, 11% requiring secondary care and follow-up care and 3% needing emergency care. Johnson et al. assessed the utilisation of teleophthalmology in a rural location using a prospective audit. They found that 31 different eye conditions were well managed using teleophthalmology [76]. This ensured access to patients in rural and remote locations and was seen as a supplement to outreach programs. Hall et al. reported on the benefit of telementoring between a metropolitan and rural hospitals to conduct ophthalmological procedures [77]. Two successful surgeries were conducted remotely, indicating that telehealth can be useful for training purposes in real-time and improve the quality of eye health services accessible by remote communities. Similarly, McGlacken-Byrne et al. reported that the addition of telehealth services improved access to surgery by reducing waiting times in a retrospective clinical audit [78]. While studies have illustrated the utility of teleophthalmology, other studies have also confirmed it is both satisfactory for patients and cost-effective. For example, a study by Host et al. evaluated patient satisfaction in 109 participants in a rural location who underwent teleophthalmology consultations [79]. Satisfaction rates were high, with 94% of participants reporting that they were either very satisfied or satisfied, and no one reported being dissatisfied or very dissatisfied [79]. Kumar et al. in 2006 also investigated the cost-effectiveness of teleophthalmology and whether it is a sustainable approach to providing eye health services [80]. The results showed that the variable costs per patient were AUD 166.89 per patient, with alternatives costing AUD 445.96, AUD 271.48 and AUD 665.44 per patient depending on the clinical scenario [80]. Teleophthalmology was calculated to have a set-up cost of AUD 13,340, and the cut-off point at which teleophthalmology became cheaper to operate compared to any of the alternative options was at a workload of 128 patients. Razavi et al. also performed a cost analysis of teleophthalmology using both retrospective and prospective audits and reported on the potential cost-saving benefit of teleophthalmology [81]. They showed that up to 15% and 24% of emergency patient transfer and outreach consultations, respectively, were suitable for teleophthalmology. These results further confirm that teleophthalmology serves as a sustainable substitute for conventional eye-care services in underserviced areas.

### 3.4. Ophthalmology Workforce Demographics

Four reports were identified from the review discussing the ophthalmology workforce [3,82,83,84] (Table 5). Two of these studies outlined both the size and distribution of the ophthalmology workforce, which highlighted the gross maldistribution towards metropolitan centres of Australia [3,82]. The 2016 Australia Future Workforce Report provided details on the distribution of the ophthalmology workforce [3]. Overall, there was a total of 985 qualified ophthalmologists and trainees recorded, representing 1050 full-time equivalent personnel. Of this number, 830 personnel worked in metropolitan cities, 73 in large regional towns and only 82 across various rural and remote areas [3]. In a more recent study by Allen et al., a high degree of location stability was also observed in the ophthalmology workforce [82]. The study analysed the practise locations of 948 ophthalmologists over a six-year period using the Australian Health Practitioner Registration Agency (AHPRA) data and found that 84% of ophthalmologists working in a metropolitan city and 79% of those working outside of metropolitan areas remained in their respective locations over the study period [82].

The other two workforce studies highlighted the increasing disparity between male and female ophthalmologist workforce participation [83,84]. Lo et al. investigated the differences in clinical practise between male and female ophthalmologists in Australia [83]. The participants were ophthalmologists who undertook the RANZCO workforce survey, the Medicine in Australia: Balancing Employment and Life survey, or those who made claims from the Medicare Benefits Schedule. The results showed that female ophthalmologists provided 35% fewer services per ophthalmologist per year, worked fewer hours and received about half the annual income of male ophthalmologists. Additionally, most female ophthalmologists reported practicing in medical subspecialties, while most male ophthalmologists were in a surgical subspecialty. In a study by Danesh et al. that included ophthalmologists from both Australia and New Zealand, similar findings were reported [84]. Not only did female ophthalmologists work fewer hours and earn less, but the results also showed that female ophthalmologists were more likely to practise in metropolitan settings compared to males and were less likely to be in a stable relationship or have children. These differences appeared not to affect career satisfaction, as no variation was observed between male and female ophthalmologists.

### 3.5. Ophthalmology Workforce Education and Training for Rural and Remote Practise

Only three studies were identified in the review that specifically discussed aspects of workforce education and training for future rural and remote practise [3,85,86] (Table 6). Firstly, the Australia Future Workforce Report provided insight into the potential future ophthalmology workforce by reporting on the characteristics of 325 hospital non-specialists with the intention of undertaking vocational ophthalmology training [3]. Fifty-six per cent were resident medical officers, 22% were females, 45% were between the age of 25 and 34 years and 53% lived in either New South Wales or Victoria [3]. Another study by Creed et al. also focused on factors likely to influence the choice of future medical specialty among medical students. Creed et al. investigated the specialty choice of 530 medical students based on their prestige and lifestyle friendliness ranking of 19 medical specialties [85]. The results showed that significant variability exists in terms of students’ preferences. In their study, ophthalmology was ranked seventh out of 19 specialties for both prestige and lifestyle friendliness. However, current evidence suggests that ophthalmology is a sought-after specialty as it is one of only two medical specialties with annual average earnings of more than half a million dollars [87].

The final study by McGrail et al. assessed the selection criteria used by 14 medical specialist colleges to determine suitable candidates for vocational training [87]. This study found that most medical specialist colleges do not have any selection criteria related to rurality, thereby limiting the number of rurally exposed doctors in vocational training. Fortunately, RANZCO, along with five other medical specialist colleges, were reported to have some rural-focused selection criteria for trainees. Specifically, RANZCO considers the rural exposure of applicants based on their background, past education, or work experience [87].

## 4. Discussion

The major training strategy to address ophthalmology workforce maldistribution in Australia is the Specialist Training Program (STP) [11], which provides an opportunity for trainees to gain knowledge and experience in training positions located outside of tertiary centres, including private and/or public facilities in regional, rural or remote settings. The expectation is that positive training experiences in these settings outside of major metropolitan hospitals will improve future workforce distribution by encouraging trainees to practise in these locations post-qualification. Considering the importance of the STP in improving future rural ophthalmic care, the aim of this review was to examine the context of ophthalmology training and practise in rural and remote Australia and the implications for future workforce development. Overall, the findings have illustrated a wealth of literature relating to Indigenous eye health and the delivery of ophthalmology services in rural and remote areas, and yet there is a paucity of information regarding workforce distribution or the training and education of ophthalmologists for work in rural and remote locations. This is concerning given the need for evidence on workforce distribution to highlight current areas of workforce shortage and hence allow for better alignment between the development of STP training opportunities and areas of ophthalmic need.

While further policy work is needed to inform the development of further STP sites, training provided at current posts can be improved by incorporating experiences that reflect real-world ophthalmology care in rural and remote locations. This will ensure trainees are better prepared and cognisant of how best to support underserved communities when they graduate. Firstly, training must include a core focus on Indigenous eye health, given the reports on the incidence and prevalence of eye diseases have largely reported a higher prevalence of eye diseases in Indigenous Australians compared to non-Indigenous Australians. Regions with a high population of Indigenous Australians, which are often rural and remote areas, should be prioritised as training locations to allow ophthalmologists to develop cultural awareness. Further, prioritising these regions would also ensure increased access to eye health services, thus potentially reducing the prevalence of eye disease both in the local Indigenous population and nationally. Considering that Indigenous people and rural residents are more likely to present at a later stage of eye disease progression, this will also allow trainees to develop confidence and competence in surgically managing more complex patient presentations and providing relevant aftercare. Finally, training additional ophthalmologists who identify as Indigenous Australians would build on the ability to provide culturally sensitive ophthalmology care to First Nations peoples in Australia [88].

Secondly, there is a need for ophthalmology training to expose trainees to rural and remote communities who suffer from the barrier of distance and develop their appreciation of alternative methods of service delivery, including both outreach and telehealth, to address eye health inequity. The benefits of teleophthalmology have been well reported in the literature [75,76,77,78,79,80,81,89]. Collectively, the reports demonstrate that teleophthalmology services can be useful in the diagnosis and management of a wide range of eye conditions in rural areas by specialists in metropolitan locations. Teleophthalmology has, however, had a reluctant uptake due to several reasons. These include poor internet access in rural communities, poor technological literacy amongst both patients and clinicians, and clinician fear of malpractice liability [90]. However, the challenge of reliable internet connectivity can be overcome by using a combination of asynchronous image transmission and real-time consultation [89]. The COVID-19 pandemic has also accelerated the need for ophthalmologists to explore the role of teleophthalmology in clinical service delivery [90]. Capitalising on this growing acceptance of telehealth and equipping trainees with skills in teleophthalmology will no doubt greatly aid uptake and its application, specifically in rural and remote practise. Telehealth could also be useful for providing mentorship to practitioners in rural areas through connection with tertiary centres in a metropolitan location [77]. This suggests that developing telehealth competence during vocational training will not only enrich trainees’ experiences but also help improve the quality of services accessible to patients in rural areas in the longer term. In addition to telehealth, rural and remote training experiences also need to expose trainees to outreach programs. This may serve to continue the strong commitment of the profession to participating in outreach programs, as documented in the literature [67]. Frequent participation in outreach programs may be because ophthalmologists acknowledge the difficulties faced by underserved communities in accessing eye care. Continuous outreach programs in rural communities may provide sufficient rural exposure to trainees who may end up practising in such communities both in the short term and long term.

Aside from telehealth and outreach services, vocational training for ophthalmologists in metropolitan teaching hospitals typically seeks to immerse trainees in training settings that offer models of care that utilise other eye health professionals, especially optometrists and nurses. This interprofessional approach to ophthalmology care is critical to supporting service delivery in rural and remote areas where there are health workforce shortages. Further, interprofessional collaborations have been documented to be beneficial in delivering eye care in both regular clinic settings and outreach programs [69,73]. The RANZCO Vocational Training Program can be structured in a way that encourages a multidisciplinary model of care to enrich the trainee experience and provide an environment for interprofessional learning and collaboration in the overall interest of the patient and the community.

Thirdly, there needs to be a strong pipeline of medical graduates into ophthalmology training positions to ensure a continued supply of ophthalmologists in the coming decade. These graduates should be selected from rural backgrounds, if possible, to leverage off the increased likelihood of them returning to non-metropolitan areas to practise [91,92,93]. Additionally, the composition of the workforce is an important consideration that can impact the availability of ophthalmologists in rural areas. Considering that the population is ageing, the demand for ophthalmologists is likely to exceed the supply in the future. Moreover, the evidence shows that the ophthalmology workforce is ageing too [3], and female ophthalmologists tend to balance work and personal responsibilities by working fewer hours, resulting in a reduction in full-time equivalent ophthalmologists available [83,84]. This highlights the need to increase the number of trainees admitted into an ophthalmology training program to ensure that there will be enough ophthalmologists in the future to cater for specialist eye service demand.

Finally, it is acknowledged that several recommendations have already been proposed by Australia’s Future Health Workforce report to improve ophthalmology workforce development, many of which are reaffirmed by the findings of this review [3]. However, this review has identified additional considerations for ophthalmology training specifically in rural and remote areas which will enhance achieving a fit-for-purpose ophthalmic workforce in the future. Specifically, this review has identified the importance of incorporating telehealth into ophthalmology training settings; collaborating with other health workers, especially optometrists and specialist nurses in eyecare delivery; and exposing trainees to more patients of Indigenous background. This review has also highlighted the need for more research focusing on ophthalmology workforce distribution to help identify evidence-based solutions for workforce maldistribution. Ongoing and expanded support by the Australian Government Department of Health through the STP and other rural health training and research funding and policy initiatives will prove critical in achieving this.

Our review has a number of limitations. Due to the large number of articles and reports from the grey literature, we have only included relevant entries identified by cross-referencing research articles included in the review. Additionally, our review only relates to the Australian context and cannot be generalised to global ophthalmology practice.

## 5. Conclusions

With an anticipated undersupply and maldistribution of ophthalmologists in the coming decade, efforts to improve training must focus on how to build a sizeable, fit-for-purpose workforce to address eye health needs across Australia. Although there is an urgent need for research and policy action into ophthalmology training in rural areas, this review has identified several training considerations that may benefit future rural ophthalmic workforce development. Aligning the location of STP posts to areas with the greatest workforce shortage, together with adapting training positions offered to reflect real-world ophthalmology care in regional, rural and remote environments, will help improve trainee skills and experience and ultimately better prepare future eye health specialists to support eye health needs across Australia.

## Figures and Tables

**Table 1:** Inclusion and exclusion criteria.

	Inclusion	Exclusion
Year	≥2005	<2005
Language	English	All other languages
Countries	Australia	All other countries
Article type (original research)		
Context	Prevalence of eye health conditions in rural and remote Australia and (would be good background to argue the need for more ophthalmologists in regional/rural/remote areas)	Prevalence of eye health conditions in rural and remote areas in other countries
	Service access and utilisation of ophthalmology related services in regional/rural/remote areas of Australia	Service access and utilisation of ophthalmology related services in rural/remote areas of other countries
	Use of telehealth to deliver ophthalmology services (could relate to training in rural/remote areas)	Outcomes of telehealth interventions in other specialties
	Service delivery models to address rural eye health inequities	Outcomes of service delivery models in other countries
	Place based surgical/treatment outcomes (e.g., rural surgery versus metro surgery)	Outcomes of specific surgical interventions
	Rural specialist practice (e.g., barriers and enablers)	Non rural practice (barriers and enablers)
Training	Attitudes of medical graduates toward ophthalmology as a speciality	Attitudes of medical graduates toward other specialities
	Attitudes of medical graduates toward rural practice/training	Attitudes of medical graduates towards other practice/training
	Training of eye health professionals (e.g., specialist nurses)	Training of other professionals
Workforce	Workforce distribution, demographics, planning and projections for ophthalmologists	Workforce distribution, demographics, planning, projections for other specialties

**Table 2:** Indigenous Eye Health.

Author	Title	Year	Research Objective	Study Design	Participants and Sample Size	Findings
Keel et al. [13]	Prevalence and associations of presenting near-vision impairment in the Australian National Eye Health Survey	2018	To determine the prevalence and associations of presenting near vision impairment (NVI) in Indigenous and non-Indigenous Australians	National survey	3098 non-Indigenous Australians and 1738 Indigenous Australians	There was a higher prevalence of near vision impairment among Indigenous people compared with non-Indigenous Australians at 34.7% versus 21.6%
Vos et al. [14]	Contribution of vision loss to the Indigenous health gap	2012	To quantify the burden of vision loss	1694 Indigenous children and 1614 Indigenous adults over the age of 40 from 30 communities	Stratified random cluster sampling design	The prevalence of blindness and vision loss in adult Indigenous Australians has a higher prevalence of vision loss compared with estimates for the total Australian population. Blindness contributed 53% to this burden of vision loss and prevalent years lost due to disability were seven times higher among Indigenous Australians compared to the general Australian population
Foreman et al. [15]	The prevalence and cause of vision loss in Indigenous and non-Indigenous Australians	2017	To determine the prevalence and cause of vision loss in Indigenous and non-Indigenous Australians	Cross-sectional population-based survey	1738 Indigenous Australians and 3098 non-Indigenous Australians	The overall prevalence of vision loss in Australia was 6.6%. The prevalence of vision loss was 11.2% in Indigenous Australians and 6.5% in non-Indigenous Australians. Vision loss was 2.8 times more prevalent in Indigenous Australians than non-Indigenous Australians after adjusting for age and gender
Keel et al. [16]	The prevalence of visually significant cataracts in the Australian National Eye Health Survey	2019	To describe the prevalence of visually significant cataracts in Indigenous and non-Indigenous Australians	Cross-sectional population-based survey	A total of 3098 non-Indigenous Australians and 1738 Indigenous Australians	The overall weighted prevalence of visually significant cataracts was 2.7% in non-Indigenous Australians and 4.3% among Indigenous Australians
Brazionis et al. [17]	Diabetic retinopathy in a remote Indigenous primary healthcare population: A Central Australian diabetic retinopathy screening study in the Telehealth Eye and Associated Medical Services Network project	2018	To determine diabetic retinopathy prevalence and severity among Indigenous Australians	Cross-sectional study	301 Indigenous adults	A total of 47% had diabetic retinopathy, of which16.2% had sight-threatening diabetic retinopathy
Landers et al. [18]	Prevalence of pseudoexfoliation syndrome in Indigenous Australians within central Australia: The Central Australian Ocular Health Study	2012	To determine the prevalence of pseudoexfoliation syndrome within the Indigenous Australian population living in central Australia	Cross-sectional study	1884 Indigenous Australians	Pseudoexfoliation was present in one or both eyes of 4.7% of individuals, and the prevalence was observed to increase with age
Ng et al. [19]	Association of Visual Impairment and All-Cause 10-Year Mortality Among Indigenous Australian Individuals Within Central Australia The Central Australian Ocular Health Study	2018	To assess the association between visual impairment and 10-year mortality risk among the remote Indigenous Australian population	Prospective cohort study	1347 Indigenous Australians	The total all-cause mortality was 29.3% at 10 years, which varied from 21.1% among those without visual impairment to 48.5% among those with visual impairment
Chang et al. [20]	Prevalence of uveitis in Indigenous populations presenting to remote clinics of central Australia: The Central Australian Ocular Health Study	2012	To determine the prevalence of current and previous uveitis within the Indigenous population	Population-based cross-sectional study	1884 Indigenous Australians	The prevalence of anterior uveitis was 0.21%, while that of posterior uveitis was 0.59%
Chua et al. [21]	Glaucoma prevalence in Indigenous Australians	2010	To determine the prevalence of glaucoma within the Indigenous Australian population	Population-based cross-sectional study	1189 Indigenous Australians	The prevalence of glaucoma was 2.2% and only 19.3% of persons with glaucoma reported a known history of glaucoma
Dirani et al. [22]	Prevalence of trachomatous trichiasis in Australia: The National Eye Health Survey	2018	To determine the prevalence of trachomatous trichiasis in Indigenous Australians aged 40 years and older	Population-based cross-sectional study	1738 Indigenous Australians aged 40 years or older	The prevalence of trachoma appears to be decreasing in Australia. Only three (0.17%) participants had trachomatous trichiasis, with no confirmed cases of trachomatous corneal opacification
Keel et al. [23]	Prevalence of glaucoma in the Australian National Eye Health Survey	2018	To estimate the prevalence of glaucoma in Australia	Population-based cross-sectional study	3098 non-Indigenous Australians (aged 50–98 years) and 1738 Indigenous Australians (aged 40–92 years)	The weighted prevalence of glaucoma in non-Indigenous Australians and Indigenous Australians was 1.5% and 0.6%, respectively
Keel et al. [24]	The prevalence of vision loss due to ocular trauma in the Australian National Eye Health Survey	2017	To determine the prevalence of vision loss due to ocular trauma in Australia	Population-based cross-sectional study	3098 non-Indigenous Australians (aged 50–98 yrs) and 1738 Indigenous Australians (aged 40–92 years)	Residing in very remote geographical areas was associated with higher odds of vision loss from ocular trauma. 2.4 per 1000 non-Indigenous and 7.9 per 1000 Indigenous Australians have vision loss due to previous ocular trauma
Keel et al. [25]	Population-based assessment of visual acuity outcomes following cataract surgery in Australia: the National Eye Health Survey	2018	To assess the visual outcomes of cataract surgery among a national sample of non-Indigenous and Indigenous Australians	Population-based study	3098 non-Indigenous Australians and 1738 Indigenous Australians	The sampling weight-adjusted cataract surgery prevalence was 19.8% in non-Indigenous Australians and 8.2% in Indigenous Australians. Among the non-Indigenous population, poor outcomes were present in 20% of participants. Among Indigenous Australians, poor outcomes were higher in 34.1% of respondents
Keel et al. [26]	Prevalence and characteristics of choroidal nevi: the Australian National Eye Health Survey	2018	To investigate the prevalence and characteristics of choroidal nevi among non-Indiginous and Indigenous Australian adults	Population-based cross-sectional study	3098 non-Indigenous Australians and 1738 Indigenous Australians (aged 40–92 years)	In the non-Indigenous population, the weighted prevalence of choroidal nevi was 2.1%. Among Indigenous Australians aged, the weighted prevalence of choroidal nevi was 0.68%
Keel et al. [27]	The Prevalence of Diabetic Retinopathy in Australian Adults with Self-Reported Diabetes: The National Eye Health Survey	2017	To investigate the prevalence of and factors associated with diabetic retinopathy among non-Indigenous and Indigenous Australian adults with self-reported diabetes	Population-based cross-sectional study	1738 Indigenous Australians and 3098 non-Indigenous Australians	The prevalence of vision-threatening retinopathy amongst Indigenous and non-Indegenous Australians was 9.5% and 4.5%, respectively. The result showed that Indigenous Australians with longer diabetes duration and those residing in remote geographical areas are at higher risk of diabetic retinopathy
Keel et al. [28]	Prevalence of Age-Related Macular Degeneration in Australia The Australian National Eye Health Survey	2017	To examine the prevalence of age-related macular degeneration (AMD) in Australia	Population-based cross-sectional study	1738 Indigenous Australians and 3098 non-Indigenous Australians	The weighted prevalence of early AMD was 14.8% and of intermediate AMD was 10.5% among non-Indigenous Australians. In Indigenous Australians, the weighted prevalence of early AMD was 13.8% and of intermediate AMD was 5.7%
Landers et al. [29]	Prevalence and associations of diabetic retinopathy in indigenous Australians within central Australia: the Central Australian Ocular Health Study	2010	To determine the prevalence and associations of diabetic retinopathy among Indigenous Australian	Prospective cohort study	1884 Indigenous adults aged ≥20 years	Among the participants recruited, 55% had reported having diabetes. Of those with diabetes, 22.2% had varying levels of retinopathy and 7.0% had vision-threatening retinopathy
Landers et al. [30]	Prevalence and associations of cataract in Indigenous Australians within central Australia: the Central Australian Ocular Health Study	2010	To determine the prevalence and associations of cataracts within the Indigenous Australian population living in central Australia	Cross-sectional survey	1884 Indigenous Australians	Among the respondents, 22.7% had any form of cataracts in one or both eyes. 12.6% of patients had a cataract that resulted in visual acuity of worse than 6/12 in one or both eyes
Landers et al. [31]	The prevalence and causes of visual impairment in Indigenous Australians within central Australia: the Central Australian Ocular Health Study	2010	To determine the prevalence and causes of visual impairment and blindness among Indigenous Australians	Clinic-based cross-sectional study	1884 individuals aged >20 years	19.4% had bilateral visual impairment, and 2.8% had bilateral blindness. Refractive error and cataracts were identified as the main causes of bilateral visual impairment and blindness
Landers et al. [32]	Prevalence of pterygium in Indigenous Australians within central Australia: the Central Australian Ocular Health Study	2011	To determine the prevalence of pterygium within the Indigenous Australian population living in central Australia	Clinic-based cross-sectional study	1884 individuals	Pterygium was present in one or both eyes of 9.3% of individuals aged 40 years or older. Pterygium was present in a higher proportion among Indigenous Australians compared with non-Indigenous Australians
Landers et al. [33]	Incidence of visual impairment due to cataract, diabetic retinopathy and trachoma in Indigenous Australians within central Australia: the Central Australian Ocular Health Study	2013	To estimate the incidence and causes of visual impairment among the Indigenous Australian population	Prospective cohort study	1884 Indigenous individuals	The incidence of visual impairment in at least one eye was 6.6%, 1.2% and 0.7% per year for cataracts, diabetic retinopathy and trachoma, respectively. Advancing age was the main risk factor common to all three
Landers et al. [34]	Prevalence of cicatricial trachoma in an Indigenous population of Central Australia: the Central Australian Trachomatous Trichiasis Study (CATTS)	2005	To determine the prevalence of cicatricial trachoma in an Indigenous population	Sample was drawn from 16 Indigenous communities	181 patients from an outreach clinic	97 (54%) patients had trachomatous scarring, 15 patients (8%) had trichiasis and 5 patients (3%) had opacities
Sherwin et al. [35]	Prevalence of chronic ocular diseases in a Genetic Isolate: the Norfolk Island Eye Study (NIES)	2011	To investigate the prevalence and causes of blindness and low vision on Norfolk Island	Population-based study	781 people aged ≥ 15 years, equal to 62% of the permanent population	Prevalence of unilateral blindness in those aged ≥ 40 was 1.5%. The most common causes of unilateral blindness were age-related macular degeneration, amblyopia, and glaucoma
Taylor et al. [36]	Vision loss in Australia	2005	To assess the prevalence and causes of vision loss in Australia and to project these data into the future	Data synthesis from existing cohort studies	8376 community and 533 nursing home residents	The most common causes of low vision were under-corrected refractive error (62%), cataracts (14%) and age-related macular degeneration (10%). The number of people with low vision and blindness is projected to almost double by 2024
Taylor et al. [37]	Cataract in Indigenous Australians: the National Indigenous Eye Health Survey	2010	To investigate the prevalence of vision loss due to cataracts in Indigenous Australians	Stratified, random cluster sample in 30 Indigenous communities	1189 Indigenous adults	Low vision due to cataracts occurred in 2.5% and blindness in 0.59%. The prevalence of vision loss due to cataracts increased with remoteness: 2.6% in major cities, 3.8% in very remote coastal and 5.2% in very remote inland
Taylor et al. [38]	The prevalence and causes of vision loss in Indigenous Australians: the National Indigenous Eye Health Survey	2010	To determine the prevalence and causes of vision loss in Indigenous Australians	Population-based cross-sectional study	1694 Indigenous children 5–15 years and 1189 Indigenous adults >40 years	Rates of low vision were 1.5% in children and 9.4% in adults. Rates of blindness were 0.2% in children and 1.9% in adults. The principal cause of low vision in both adults and children was refractive error
Yazar et al. [39]	Raine eye health study: design, methodology and baseline prevalence of ophthalmic disease in a birth-cohort study of young adults. Ophthalmic genetics	2013	To determine the baseline prevalence of ophthalmic disease in the Western Australian pregnancy cohort study	Retrospective, observational clinical audit	1344 participants	A total of 5.5% had myopia, and 1.2% had unilateral or bilateral pterygia. Other conditionssuch as keratoconus, cataract, keratitis and uveitis were rare
Abouzeid et al. [40]	Equity in vision in Australia is in sight	2015	Report on closing the gap for vision	NA	NA	The disparities in eye health between Indigenous and non-Indigenous Australians can be eliminated with concerted political will.
Clark et al. [41]	Diabetic retinopathy and the major causes of vision loss in Aboriginals from remote Western Australia	2010	To report on diabetic retinopathy and the major causes of vision loss and blindness among Indigenous people	Prospective study	920 Indigenous people participated in 1331 examinations over the study period	The major causes of vision loss were cataract, uncorrected refractive error and trauma. Indigenous people with diabetes were far more likely to have vision loss. Among those with diabetes, 82 (24.9%) had diabetic retinopathy, and 32 (9.7%) had vision-threatening retinopathy
Estevez et al. [42]	Association of disease-specific causes of visual impairment and 10-year mortality amongst Indigenous Australians: the Central Australian Ocular Health Study	2018	To determine the association between causes of visual impairment and all-cause mortality	Retrospective cohort analysis	1347 Indigenous Australians	Among participants with visual impairment, the overall mortality rate was 45%. Participants with a visual impairment from diabetic retinopathy were more likely to die during follow-up compared with those without visual impairment
Foreman et al. [43]	Treatment coverage rates for refractive error in the National Eye Health survey	2017	To determine treatment coverage rates and risk factors associated with uncorrected refractive errors	1738 Indigenous Australians 3098 non-Indigenous Australians	1738 Indigenous Australians and 3098 non-Indigenous Australians	The refractive error treatment coverage rate in Indigenous Australians was 82.2% compared to 93.5% in non-Indigenous Australians. In Indigenous participants, remoteness, having never undergone an eye examination and having consulted a health worker other than an optometrist or ophthalmologist were risk factors for low coverage
Foreman et al. [44]	Cataract surgery coverage rates for Indigenous and non-Indigenous Australians: the National Eye Health Survey	2017	To determine cataract surgery coverage rates for Indigenous and non-Indigenous Australians	Nationwide, cross-sectional population-based survey	1738 Indigenous Australians and 3098 non-Indigenous Australians	Cataract surgery coverage rates were lower for Indigenous Australians at 58.5% compared to non-Indigenous participants at 88.0%
Keel et al. [45]	Participant referral rate in the National Eye Health Survey (NEHS)	2017	To present the rates of referral of participants in the National Eye Health Survey (NEHS) for further eye care	Australia that each included about 300 Indigenous	3098 non-Indigenous Australians and 1738 Indigenous Australians	A total of 32.1% of non-Indigenous participants and 43.6% of Indigenous participants were referred for further eye care. Among Indigenous participants, there were proportions of referrals to remote and very remote areas compared to major cities
Landers et al. [46]	Prevalence and associations of refractive error in Indigenous Australians within central Australia: the Central Australian Ocular Health Study	2010	To determine the prevalence and associations of refractive error within the Indigenous Australian population living in Central Australia	Prospective observational cohort study	1884 participants	A total of 15.2% were hypermetropic, 11.1% were myopic, and 6.2% had astigmatism. Participants became progressively more hypermetropic with increasing age until the age of 70 years, after which time they become more myopic.
Landers et al. [47]	Presence of diabetic retinopathy is associated with worse 10-year mortality among Indigenous Australians in Central Australia: The Central Australian ocular health study	2019	To investigate associations between 10-year mortality and the presence of diabetes among Indigenous Australians	Prospective observational cohort study	1257 participants	Ten-year all-cause mortality was found to be 29.3%. Of those with diabetes mellitus but no retinopathy, 24.0% died during the 10 years after recruitment, compared with 40.1% for those with any retinopathy. Those who had any retinopathy were 75% more likely to die.
Liu et al. [48]	Ten-year all-cause mortality and its association with vision among Indigenous Australians within Central Australia: the Central Australian Ocular Health Study	2016	To identify the extent of ocular morbidity of Indigenous Australians in 30 rural and remote communities in Central Australia	Prospective observational cohort study	1257 participants	Reduced visual acuity was significantly associated with an increased mortality rate, with a 5% increased mortality per one line of reduced visual acuity after adjusting for age, gender, diabetes and hypertension
Taylor et al. [49]	Projected Needs for Eye Care Services for Indigenous Australians	2011	To identify the estimated needs for eye care services among Indigenous Australians	Data retrieved from the Australian Bureau of Statistics (ABS) Census 2006	455,027 participants	Compared to the rest of the population, unmet needs for the provision of eye care services existed among Indigenous Australians
Taylor et al. [50]	Provision of Indigenous Eye Health Services	2010	To determine the degree, distribution and causes of vision loss among Indigenous people	A randomized multi-stage cluster survey in 30 sites nationally	1694 children and 1189 adults	Blindness rates in Indigenous adults were 6 times more compared to the rest of the population, and 35% of Indigenous adults have never had an eye examination. There is a marked shortage of ophthalmic services in more remote and very remote communities.
Wright et al. [51]	Trachoma, cataracts and uncorrected refractive error are still important contributors to visual morbidity in two remote Indigenous communities of the Northern Territory, Australia	2009	To assess the contribution of trachoma, cataract, and refractive error to visual morbidity among Indigenous adults living in two remote communities	Cross-sectional survey of all adults aged 40 and over within a desert and coastal community	260 participants	The prevalence of visual impairment was 17%. In total, 78% of adults who grew up in a desert community had trachomatous scarring compared with 26% of those who grew up in a coastal community. In the desert community, the prevalence of trachomatous trichiasis was 10% and corneal opacity was 6%
Brazionis et al. [52]	Associations with sight-threatening diabetic macular oedema among Indigenous adults with type 2 diabetes attending an Indigenous primary care clinic in remote Australia: a Centre of Research Excellence in Diabetic Retinopathy and Telehealth Eye and Associated Medical Services Network study	2021	To identify factors associated with sight-threatening diabetic macular oedema in Indigenous Australians attending a primary care centre	Cross-sectional screening study	236 adult participants	The prevalence of sight-threatening diabetes was 14.8% and similar in men and women and was associated with longer diabetes duration and markers of renal impairment
Foreman et al. [53]	Future burden of vision loss in Australia: Projections from the National Eye Health Survey	2020	This study aimed to forecast bilateral vision loss in Australia from 2020 to 2050	Population-based survey	4523 Indigenous and non-Indigenous Australians	The prevalence of vision loss is likely to increase from 6.7% to 7.5% by 2050. The estimated number of Australians ≥50 years old with vision loss will nearly double from 532,386 in 2016 to 1 ,015,021 by 2050
Quinn et al. [54]	Screening for diabetic retinopathy and reduced vision among Indigenous Australians in Top End primary care health services: A TEAMS net sub-study	2020	To investigate the prevalence of diabetic retinopathy, reduced vision and diabetes retinopathy treatment among Indigenous Australian adults	Cross-sectional screening study in two Aboriginal primary healthcare services	287 participants	The prevalence of non-proliferative and proliferative retinopathy was 37.3% and 5.4%, respectively, and 9.0%, respectively, for one site. The proportion with normal vision, reduced vision, impaired vision and blindness was 31.1%, 52.5%,15.6% and 0.8%, respectively

**Table 3:** Service access and utilisation of ophthalmology related service in rural and remote areas.

Author	Title	Year	Research Objective	Study Design	Participants and Sample Size	Findings
Foreman et al. [55]	Utilisation of eye health-care services in Australia: the National Eye Health Survey	2018	To investigate the utilization of eye health-care services by Australians	Cross-sectional survey	1738 Australians and 3098 non-Indigenous Australians	About 67.0% of Indigenous Australians and 82.5% of non-Indigenous Australians underwent an eye examination within the previous 2 years. Indigenous status, male gender, living in outer regional and very remote residences were associated with less recent examinations
Boudville et al. [56]	Indigenous access to cataract surgery: an assessment of the barriers and solutions within the Australian health system	2013	To identify barriers within the health systems that limit access to cataract surgery for Indigenous Australians	Interview and focus group-based qualitative study	530 participants	Several barriers were identified, including long waiting times, health system complexities, cost of surgery, lack of surgical capacity and limited awareness of regional eye health needs
Arnold et al. [57]	Use of eye care services by Indigenous Australian adults	2011	Investigation of access to eye care services by Indigenous Australians	Survey-National Indigenous Eye Health Survey	1694 Indigenous children and 1189 Indigenous adults from 30 communities across Australia	Barriers to seeking treatment included lack of services in their area and transport or distance issues
Turner et al. [58]	Eye health service access and utilization in the National Indigenous Eye Health Survey	2011	To determine access to and utilization of eye health services for Indigenous Australians	National wide stratified random sampling	1189 Indigenous adults	Uncorrected refractive error caused 54% of the low vision in more than half of the study’s participants and 14% of blindness. Cataract surgical services were located in a number of study sites and patients were required to fly for surgery in other sites
Muller et al. [59]	Changes in eye care utilization following an eye health promotion campaign	2010	To describe changes in eye care services utilization after a public health campaign	Longitudinal study	1695 people participated in the baseline and 1728 people in the follow-up study	The percentage of people who visited an eye specialist increased significantly from 61% to 70%. Additionally, the percentage of those with diabetes that reported having a dilated fundus examination within the last 2 years increased significantly from 52% to 70%
Wright et al. [60]	Barriers to the Implementation of the SAFE Strategy to Combat Hyperendemic Trachoma in Australia	2010	To identify some of the barriers to the implementation of the trachoma management strategy within remote Indigenous communities	Qualitative study using semi-structured interviews	14 healthcare professionals	A number of barriers were identified, including lack of finance, workforce and community awareness
Joyce et al. ^b^ [61]	Adoption, implementation and prioritization of specialist outreach policy in Australia: a national perspective	2014	To describe the adoption, implementation and prioritization of a national specialist outreach policy in Australia	Policy discussion	NA	To be successful, outreach policy must harmonize with the interests of the workforce and support professional autonomy
Pearse et al. [62]	Outreach and improved access to specialist services for Indigenous people in remote Australia: the requirements for sustainability	2002	To examine the role of specialist outreach in supporting primary health care and overcoming the barriers to health care faced by the indigenous population in remote areas of Australia and to examine issues affecting its sustainability	Process evaluation of a specialist outreach service, using health service utilisation data and interviews with health professionals and patients	17 remote health practitioners, five specialists undertaking outreach, five regional health administrators, and three patients from remote communities	Outreach delivery of specialist services has overcome some of the barriers relating to distance, communication and cultural inappropriateness of services and has enabled an over four-fold increase in the number of consultations with people from remote communities
Boudville et al. [63]	Improving eye care for Indigenous Australians in primary health care settings	2013	To assess the barriers and solutions to the delivery of eye care in primary care settings and solutions to improve the use of comprehensive eye care among Indigenous Australians	Qualitative study	289 participants in a semi-structured interview and 116 in a stakeholders’ workshop	The costs associated with consultations, the cost of spectacles and other specialist fees were reported as significant barriers. Enablers included the use of prompts for primary care providers and the inclusion of eye care items on health forms
Copeland et al. [64]	Understanding Indigenous patient attendance: A qualitative study	2017	To investigate the reasons for Indigenous patient non-attendance at medical specialty appointments	Qualitative study using face-to-face semi-structured interviews	69 Indigenous Australian patients and 8 clinic workers	Improving health literacy and supportive clinic staff were useful in motivating reluctant patients to attend eye care appointments
Finger et al. [65]	Disparities in access to anti-vascular endothelial growth factor treatment for neovascular age-related macular degeneration	2016	To assess eye treatment provision for late neovascular age-related macular degeneration	Analysis Medicare Australia, RANZCO, Optometry Australia, the Blue Mountains Eye Study and the ABS data	327,390 patients	There were about 7316 incidents cases of untreated cases per year. A lower number of ophthalmologists and optometrists and being located in remote regions were associated with a higher percentage of untreated cases
Taylor et al. ^a^ [66]	Cataract surgical blitzes: an Australian anachronism	2015	Discussion on the need to develop sustainable local eye health services	Report	NA	Surgical blitzes may achieve short-term gains, but they inhibit the development of sustainable local services

^a^ policy paper, ^b^ report.

**Table 4:** Service delivery models in rural and remote areas.

Author	Title	Year	Research Objective	Study Design	Participants and Sample Size	Findings
O’Sullivan et al. [67]	Rural outreach by specialist doctors in Australia: a national cross-sectional study of supply and distribution	2014	To investigate the proportion of Australian specialist doctors who participate in rural outreach service provision	Survey	4596 specialists	A total of 19% provide specialist services. Ophthalmologists are among the top five medical specialities providing outreach services
Fu et al. [68]	Evaluating the impact of the Lions Outback Vision mobile ophthalmology service	2019	To evaluate the impact of the Lions Outback Vision Van	Study evaluation	16 regional towns	A total of 16 regional towns were visited at least twice per year, with a travel coverage of up to 25,000 kilometres. The service augmented existing outreach services
Turner et al. [69]	Funding models for outreach ophthalmology services	2011	To describe funding models used in outreach eye services and their impact on clinical activity	Semi-structured interviews	Several health professionals were interviewed	Fee-for-service funding was found to increase clinical activity compared to the salary model
Glasson et al. [70]	An Innovative Australian Outreach Model of Diabetic Retinopathy Screening in Remote Communities	2015	To compare the proportion of remote diabetic patients receiving appropriate diabetic retinopathy screening prior to and following implementation of the service	Retrospectivedescriptive study	141 patients	The outreach model improved accessibility to diabetic retinopathy screening in remote communities. Of the 141 patients who underwent diabetic retinopathy screening, 16.3% had received appropriate diabetic retinopathy screening prior to the implementation of the service. After implementation, 66.3% of patients underwent appropriate screening
Glasson et al. [71]	What do patients with diabetes and providers think of an innovative Australian model of remote diabetic retinopathy screening? A qualitative study	2017	To explore the acceptability of a diabetic retinopathy screening model	Qualitative study used semi-structured interviews with patients	14 patients and 9 health professionals or stakeholders	There was improved access to diabetic retinopathy screening, and screening was highly acceptable to patients and health professionals
Slight et al. [72]	The impact of a glaucoma nurse specialist role on glaucoma waiting lists	2009	To investigate if there is a reduction in waiting list numbers and length of time waiting for after the introduction of a clinical nurse specialist clinic	Clinical audit	300 patients	The introduction of the glaucoma clinical nurse specialist led to a reduction in waiting lists and has facilitated the care of a wide range of patients, including those with complex requirements
O’Connor et al. [73]	Shared care for chronic eye diseases: perspectives of ophthalmologists, optometrists and patients	2012	To report the perspectives of optometrists, ophthalmologists and patients on a model of shared care for patients with chronic eye diseases	Qualitative study	5 ophthalmologists, 11 optometrists and 37 patients were interviewed	Optometrists met and exceeded ophthalmologists’ expectations by appropriately detecting and referring patients with additional, previously undetected conditions. Patients reported savings in travel time and were satisfied with the quality of care they received
Turner et al. [74]	Coordination of outreach eye services in remote Australia	2011	To describe models for service integration between ophthalmology and optometry when conducting outreach eye services	Semi-structured interviews	Nine selected outreach ophthalmology services	Service integration between optometry and ophthalmology showed an increased surgical case rate for ophthalmology clinics with a trend towards increased clinical activity and reduced waiting times
Kumar et al. [75]	Emergency eye care in rural Australia: role of internet	2006	To demonstrate, how an internet-based service’s impact on emergency eye care in rural Australia	Service evaluation	118 persons took part in teleophthalmology consultations	Teleophthalmology service was utilized for primary eye care (86%), secondary and follow-up care (11%) and emergency cases (3%), with an average time of telemedicine consultation was 30 min per patient
Johnson et al. [76]	Real-time teleophthalmology in rural Western Australia	2015	To assess the current utilisation of a real-time teleophthalmology service for rural Western Australia (WA)	Service evaluation by prospective audit	85 patients from rural Western Australia	Real-time teleophthalmology was used in the management of a broad range of eye conditions and was a useful supplement to outreach ophthalmology services
Hall et al. [77]	Teleophthalmology-Assisted Corneal Foreign Body Removal in a Rural Hospital	2005	To describe the use of telementoring from a metropolitan hospital to a remote hospital	Case report	2 cases	The case studies showed how telemedicine technology can be utilised for skill transfer. It demonstrated the effectiveness, safety, and economy of this service to support rural eye health care efforts
MacGlacken-Byrne et al. [78]	Review of cataract surgery in rural north Western Australia with the Lions Outback Vision	2017	To address geographic barriers using a teleophthalmology initiative to provide care to patients in regional areas	Retrospective observational clinical audit	315 patients	The addition of telehealth services improved access to surgery by reducing waiting times significantly
Host et al. [79]	Real-time teleophthalmology video consultation: an analysis of patient satisfaction in rural Western Australia	2018	To evaluate patient satisfaction with teleophthalmology	Patient survey	109 patients who underwent a video consultation with Lions Outback Vision	The most common diagnosis was cataract, followed by glaucoma and retinal disorders. The majority of the participants were either ‘very satisfied’ (69.1 per cent) or ‘satisfied’ (24.5 per cent) with the service
Kumar et al. [80]	Remote ophthalmology services: cost comparison of telemedicine and alternative service delivery options	2006	To evaluate the cost of a teleophthalmology service from a healthcare service provider’s perspective and compare it with the cost of alternatives	Cost analysis	Data relating to the costs were obtained from a number of databases, including the Commonwealth Medicare Benefits Schedule	The variable costs of teleophthalmology were UD 166.89 (Australian dollars) per patient, and the alternatives cost AUD 445.96, AUD 271.48 and AUD 665.44 per patient. Teleophthalmology offers a viable alternative to conventional eye-care services in rural and remote areas
Razavi et al. [81]	Increasing the impact of teleophthalmology in Australia: Analysis of structural and economic drivers in a state service	2017	To perform a cost analysis of teleophthalmology in Western Australia, and sought to identify efficient models of service delivery	Retrospective and prospective hospital-based clinical audits	5456 patients	A total of 15% and 24% of urgent patient transfers and outreach consultations, respectively, were found to be suitable for teleophthalmology. Teleophthalmology can lead to a potential cost saving of AUD 1.1 million/year

**Table 5:** Ophthalmology workforce demographics.

Author	Title	Year	Research Objective	Study Design	Participants and Sample Size	Findings
Department of Health Australia ^b^ [3]	Australia’s Future Health Workforce	2018	Report on the ophthalmology workforce	Survey	985 specialists and trainees	There were 985 specialists and trainees in Australia in 2015. Of this number, 830 personnel worked in a metropolitan city, 73 in large regional towns and only 82 across various categories of rural and remote areas
Allen et al. [82]	Distribution and Location Stability of the Australian Ophthalmology Workforce: 2014–2019	2021	To investigate the ophthalmology workforce distribution and location stability using the Modified Monash Model category of remoteness	Retrospective cohort study	948 ophthalmologists	Eighty-four per cent of those in aa metropolitan area remained in these areas over the six years study period. Similarly, 79% of those working outside of metropolitan areas remained in non-metropolitan areas over the six years study period
Lo et al. [83]	Differences in practice of ophthalmology by gender in Australia	2019	To determine the differences in clinical practice between female and male ophthalmologists in Australia	Cross-sectional study	91 ophthalmologists	Female ophthalmologists provided fewer services compared to males. They also received only about half the annual income of male ophthalmologists
Danesh-meyer et al. [84]	Differences in practice and personal profiles between male and female ophthalmologists	2007	To assess practice profiles and attitudes towards career and family among ophthalmologists in Australia and New Zealand, with an emphasis on identifying gender differences	Survey	254 ophthamologists	Female ophthalmologists work fewer hours and earn less. They were also more likely to practice in metropolitan settings compared to males and less likely to be in a stable relationship or have children

^b^ report.

**Table 6:** Ophthalmology workforce education and training for rural and remote practise.

Author	Title	Year	Research Objective	Study Design	Participants and Sample Size	Findings
Department of Health Australia ^b^ [3]	Australia’s Future Health Workforce	2018	Report on the ophthalmology workforce	Survey	985	Of the 325 hospital non-specialists with the intention to undertake vocational ophthalmology training, 56% were resident medical officers, 22% were females, 45% were between the age of 25 and 34 years and 53% live in either New South Wales or Victoria
Creed et al. [85]	Medical specialty prestige and lifestyle preferences for medical students	2010	To investigate the lifestyle friendliness and prestigiousness ranking of medical specialty by medical students	Survey	530 completed the prestigiousness ranking, while 644 completed the lifestyle friendliness ranking	The results showed that significant variability exists in terms of students’ preferences. Ophthalmology ranked seventh out of 19 specialties for both prestige and lifestyle friendliness
McGrail et al. [87]	Critically reviewing the policies used by colleges to select doctors for specialty training: A kink in the rural pathway	2020	To assess the selection criteria used by speciality colleges for vocational training	A systematic desk audit of colleges selection criteria	14 medical specialist colleges	Only six colleges had some rural-focused selection criteria, including RANZCO and the Australian College of Rural and Remote Medicine. RANZCO considers the rural exposure of the applicants based on their background, schooling or work experience

^b^ report.

## References

[1] Australian Institute of Health and Welfare Eye Health Treatment and Management. https://www.aihw.gov.au/reports/eye-health/eye-health/contents/treatment-and-management.

[2] Department of Health (2019). National Medical Workforce Strategy Scoping Framework. https://www.health.gov.au/sites/default/files/documents/2021/09/national-medical-workforce-strategy-scoping-framework.pdf.

[3] Department of Health (2018). Australia’s Future Health Workforce–Ophthalmology. https://www.health.gov.au/resources/publications/ophthalmology-australias-future-health-workforce-report.

[4] Department of Health (2012). Health Workforce 2025 Doctors, Nurses and Midwives. https://www1.health.gov.au/internet/publications/publishing.nsf/Content/work-review-australian-government-health-workforce-programs-toc~appendices~appendix-ii-health-workforce-2025-summary.

[5] Caffery L.J., Taylor M., Gole G., Smith A.C. (2019). Models of care in tele-ophthalmology: A scoping review. J. Telemed. Telecare.

[6] Department of Health (2015). Discussion Paper: Review of the Specialist Training Programme. https://www1.health.gov.au/internet/main/publishing.nsf/Content/review_specialist_training_program_stp.

[7] Department of Health Specialist Training Program (STP) Operational Framework 2022–2025. https://www.health.gov.au/resources/publications/specialist-training-program-operational-framework.

[8] Colquhoun H.L., Levac D., O’Brien K.K., Straus S., Tricco A.C., Perrier L., Kastner M., Moher D. (2014). Scoping reviews: Time for clarity in definition, methods, and reporting. J. Clin. Epidemiol..

[9] Arksey H., O’Malley L. (2005). Scoping studies: Towards a methodological framework. Int. J. Soc. Res. Methodol..

[10] Tricco A.C., Lillie E., Zarin W., O’Brien K.K., Colquhoun H., Levac D., Moher D., Peters M.D., Horsley T., Weeks L. (2018). PRISMA extension for scoping reviews (PRISMA-ScR): Checklist and explanation. Ann. Intern. Med..

[11] The Royal Australian and New Zealand College of Ophthalmologists (2020). Community Engagement. https://ranzco.edu/home/community-engagement/.

[12] Thomas J., Harden A. (2008). Methods for the thematic synthesis of qualitative research in systematic reviews. BMC Med. Res. Methodol..

[13] Keel S., Foreman J., Xie J., Taylor H.R., Dirani M. (2018). Prevalence and associations of presenting near-vision impairment in the Australian National Eye Health Survey. Eye.

[14] Vos T., Taylor H.R. (2012). Contribution of vision loss to the Indigenous health gap. Clin. Exp. Ophthalmol..

[15] Foreman J., Xie J., Keel S., van Wijngaarden P., Sandhu S.S., Ang G.S., Gaskin G.F., Crowston J., Bourne R., Taylor H.R. (2017). The Prevalence and Causes of Vision Loss in Indigenous and Non-Indigenous Australians: The National Eye Health Survey. Ophthalmology.

[16] Keel S., McGuiness M.B., Foreman J., Taylor H.R., Dirani M. (2019). The prevalence of visually significant cataract in the Australian National Eye Health Survey. Eye.

[17] Brazionis L., Jenkins A., Keech A., Ryan C., Brown A., Boffa J., Bursell S., on behalf of the CRE in Diabetic Retinopathy and the TEAMSnet Study Group (2018). Diabetic retinopathy in a remote Indigenous primary healthcare population: A Central Australian diabetic retinopathy screening study in the Telehealth Eye and Associated Medical Services Network project. Diabet. Med..

[18] Landers J., Henderson T., Craig J. (2012). Prevalence of pseudoexfoliation syndrome in indigenous Australians within central Australia: The Central Australian Ocular Health Study. Clin. Exp. Ophthalmol..

[19] Ng S.K., Kahawita S., Andrew N.H., Henderson T., Craig J.E., Landers J. (2018). Association of Visual Impairment and All-Cause 10-Year Mortality among Indigenous Australian Individuals within Central Australia: The Central Australian Ocular Health Study. JAMA Ophthalmol..

[20] Chang J.H., Landers J., Henderson T.R., Craig J.E. (2012). Prevalence of uveitis in Indigenous populations presenting to remote clinics of central Australia: The Central Australian Ocular Health Study. Clin. Exp. Ophthalmol..

[21] Chua B.E., Xie J., Arnold A.L., Koukouras I., Keeffe J.E., Taylor H.R. (2011). Glaucoma prevalence in Indigenous Australians. Br. J. Ophthalmol..

[22] Dirani M., Keel S., Foreman J., van Wijngaarden P., Taylor H.R. (2018). Prevalence of trachomatous trichiasis in Australia: The National Eye Health Survey. Clin. Exp. Ophthalmol..

[23] Keel S., Xie J., Foreman J., Lee P.Y., Alwan M., Fahy E.T., van Wijngaarden P., Gaskin G.C.F., Ang G.S., Crowston J.G. (2019). Prevalence of glaucoma in the Australian National Eye Health Survey. Br. J. Ophthalmol..

[24] Keel S., Xie J., Foreman J., Taylor H.R., Dirani M. (2017). The prevalence of vision loss due to ocular trauma in the Australian National Eye Health Survey. Injury.

[25] Keel S., Xie J., Foreman J., Taylor H.R., Dirani M. (2018). Population-based assessment of visual acuity outcomes following cataract surgery in Australia: The National Eye Health Survey. Br. J. Ophthalmol..

[26] Keel S., Xie J., Foreman J., Taylor H.R., Dirani M. (2018). Prevalence and characteristics of choroidal nevi: The Australian National Eye Health Survey. Clin. Exp. Ophthalmol..

[27] Keel S., Xie J., Foreman J., van Wijngaarden P., Taylor H.R., Dirani M. (2017). The Prevalence of Diabetic Retinopathy in Australian Adults with Self-Reported Diabetes: The National Eye Health Survey. Ophthalmology.

[28] Keel S., Xie J., Foreman J., van Wijngaarden P., Taylor H.R., Dirani M. (2017). Prevalence of Age-Related Macular Degeneration in Australia: The Australian National Eye Health Survey. JAMA Ophthalmol..

[29] Landers J., Henderson T., Abhary S., Craig J. (2010). Prevalence and associations of diabetic retinopathy in Indigenous Australians within central Australia: The Central Australian Ocular Health Study. Clin. Exp. Ophthalmol..

[30] Landers J., Henderson T., Craig J. (2010). Prevalence and associations of cataract in indigenous Australians within central Australia: The Central Australian Ocular Health Study. Clin. Exp. Ophthalmol..

[31] Landers J., Henderson T., Craig J. (2010). The prevalence and causes of visual impairment in Indigenous Australians within central Australia: The Central Australian Ocular Health Study. Br. J. Ophthalmol..

[32] Landers J., Henderson T., Craig J. (2011). Prevalence of pterygium in Indigenous Australians within central Australia: The Central Australian Ocular Health Study. Clin. Exp. Ophthalmol..

[33] Landers J., Henderson T., Craig J.E. (2013). Incidence of visual impairment due to cataract, diabetic retinopathy and trachoma in Indigenous Australians within central Australia: The Central Australian Ocular Health Study. Clin. Exp. Ophthalmol..

[34] Landers J., Kleinschmidt A., Wu J., Burt B., Ewald D., Henderson T. (2005). Prevalence of cicatricial trachoma in an Indigenous population of Central Australia: The Central Australian Trachomatous Trichiasis Study (CATTS). Clin. Exp. Ophthalmol..

[35] Sherwin J.C., Kearns L.S., Hewitt A.W., Ma Y., Kelly J., Griffiths L.R., Mackey D.A. (2011). Prevalence of chronic ocular diseases in a genetic isolate: The Norfolk Island Eye Study (NIES). Ophthalmic Epidemiol..

[36] Taylor H.R., Keeffe J.E., Vu H.T., Wang J.J., Rochtchina E., Mitchell P., Pezzullo M.L. (2005). Vision loss in Australia. Med. J. Aust..

[37] Taylor H.R., Xie J., Arnold A.L., Goujon N., Dunn R.A., Fox S., Keeffe J. (2010). Cataract in Indigenous Australians: The National Indigenous Eye Health Survey. Clin. Exp. Ophthalmol..

[38] Taylor H.R., Xie J., Fox S., Dunn R.A., Arnold A.L., Keeffe J.E. (2010). The prevalence and causes of vision loss in Indigenous Australians: The National Indigenous Eye Health Survey. Med. J. Aust..

[39] Yazar S., Forward H., McKnight C.M., Tan A., Soloshenko A., Oates S.K., Ang W., Sherwin J.C., Wood D., Mountain J.A. (2013). Raine eye health study: Design, methodology and baseline prevalence of ophthalmic disease in a birth-cohort study of young adults. Ophthalmic Genet..

[40] Abouzeid M., Anjou M.D., Taylor H.R. (2015). Equity in vision in Australia is in sight. Med. J. Aust..

[41] Clark A., Morgan W.H., Kain S., Farah H., Armstrong K., Preen D., Semmens J.B., Yu D.-Y. (2010). Diabetic retinopathy and the major causes of vision loss in Aboriginals from remote Western Australia. Clin. Exp. Ophthalmol..

[42] Estevez J., Kaidonis G., Henderson T., Craig J.E., Landers J. (2018). Association of disease-specific causes of visual impairment and 10-year mortality amongst Indigenous Australians: The Central Australian Ocular Health Study. Clin. Exp. Ophthalmol..

[43] Foreman J., Xie J., Keel S., Taylor H.R., Dirani M. (2017). Treatment coverage rates for refractive error in the National Eye Health survey. PLoS ONE.

[44] Foreman J., Xie J., Keel S., van Wijngaarden P., Crowston J., Taylor H.R., Dirani M. (2017). Cataract surgery coverage rates for Indigenous and non-Indigenous Australians: The National Eye Health Survey. Med. J. Aust..

[45] Keel S., Lee P.Y., Foreman J., van Wijngaarden P., Taylor H.R., Dirani M. (2017). Participant referral rate in the National Eye Health Survey (NEHS). PLoS ONE.

[46] Landers J., Henderson T., Craig J. (2010). Prevalence and associations of refractive error in Indigenous Australians within central Australia: The Central Australian Ocular Health Study. Clin. Exp. Ophthalmol..

[47] Landers J., Liu E., Estevez J., Henderson T., Craig J.E. (2019). Presence of diabetic retinopathy is associated with worse 10-year mortality among Indigenous Australians in Central Australia: The Central Australian ocular health study. Clin. Exp. Ophthalmol..

[48] Liu E., Ng S.K., Kahawita S., Andrew N.H., Henderson T., Craig J.E., Landers J. (2017). Ten-year all-cause mortality and its association with vision among Indigenous Australians within Central Australia: The Central Australian Ocular Health Study. Clin. Exp. Ophthalmol..

[49] Brando A., Hsueh Y.S., Dunt D., Stanford E., Taylor H.R. (2011). Projected needs for eye-care services in indigenous Australians. Clin. Exp. Ophthalmol..

[50] Taylor H.R., Stanford E. (2010). Provision of Indigenous Eye Health Services.

[51] Wright H.R., Keeffe J.E., Taylor H.R. (2009). Trachoma, cataracts and uncorrected refractive error are still important contributors to visual morbidity in two remote Indigenous communities of the Northern Territory, Australia. Clin. Exp. Ophthalmol..

[52] Brazionis L., Keech A., Ryan C., Brown A., O’Neal D., Boffa J., Bursell S.-E., Jenkins A. (2021). Associations with sight-threatening diabetic macular oedema among Indigenous adults with type 2 diabetes attending an Indigenous primary care clinic in remote Australia: A Centre of Research Excellence in Diabetic Retinopathy and Telehealth Eye and Associated Medical Services Network study. BMJ Open Ophthalmol..

[53] Foreman J., Keel S., McGuiness M., Liew D., van Wijngaarden P., Taylor H.R., Dirani M. (2020). Future burden of vision loss in Australia: Projections from the National Eye Health Survey. Clin. Exp. Ophthalmol..

[54] Quinn N., Yang F., Ryan C., Bursell S.E., Keech A., Atkinson-Briggs S., Jenkins A., Brazionis L., Centre of Research Excellence in Diabetic Retinopathy Study and TEAMSnet Study Groups (2020). Screening for Diabetic Retinopathy and Reduced Vision among Indigenous Australians in Top End Primary Care Health Services: A TEAMSnet Sub-study. Intern. Med. J..

[55] Foreman J., Xie J., Keel S., Taylor H.R., Dirani M. (2018). Utilization of eye health-care services in Australia: The National Eye Health Survey. Clin. Exp. Ophthalmol..

[56] Boudville A.I., Anjou M.D., Taylor H.R. (2013). Indigenous access to cataract surgery: An assessment of the barriers and solutions within the Australian health system. Clin. Exp. Ophthalmol..

[57] Arnold A.-L.M., Busija L., Keeffe J.E., Taylor H.R. (2011). Use of eye care services by Indigenous Australian adults. Med. J. Aust..

[58] Turner A.W., Xie J., Arnold A.L., Dunn R.A., Taylor H.R. (2011). Eye health service access and utilization in the National Indigenous Eye Health Survey. Clin. Exp. Ophthalmol..

[59] Muller A., Keeffe J.E., Taylor H.R. (2007). Changes in eye care utilization following an eye health promotion campaign. Clin. Exp. Ophthalmol..

[60] Wright H.R., Keeffe J.E., Taylor H.R. (2010). Barriers to the implementation of the SAFE strategy to combat hyperendemic trachoma in Australia. Ophthalmic Epidemiol..

[61] Joyce C.M., McGrail M.R. (2014). Adoption, implementation and prioritization of specialist outreach policy in Australia: A national perspective. Bull. World Health Organ..

[62] Pearse J., Mazevska D., Hachigo A. The impact of the Medical Specialist Outreach Assistance Program on improved access to specialist services for regional and remote Australia. Proceedings of the 12th National Rural Health Conference.

[63] Boudville A.I., Anjou M.D., Taylor H.R. (2013). Improving eye care for I ndigenous A ustralians in primary health care settings. Aust. J. Rural. Health.

[64] Copeland S., Muir J., Turner A. (2017). Understanding Indigenous patient attendance: A qualitative study. Aust. J. Rural. Health.

[65] Finger R.P., Xie J., Fotis K., Parikh S., Cummins R., Mitchell P., Guymer R.H. (2017). Disparities in access to anti-vascular endothelial growth factor treatment for neovascular age-related macular degeneration. Clin. Exp. Ophthalmol..

[66] Taylor H.R., Henderson T.R., Le Mesurier R.T. (2015). Cataract surgical blitzes: An Australian anachronism. Med. J. Aust..

[67] O’Sullivan B.G., Joyce C.M., McGrail M.R. (2014). Rural outreach by specialist doctors in Australia: A national cross-sectional study of supply and distribution. Hum. Resour. Health.

[68] Fu S., Jeyaraj J., Turner A.W. (2019). Evaluating the impact of the Lions Outback Vision mobile ophthalmology service. Clin. Exp. Ophthalmol..

[69] Turner A.W., Mulholland W., Taylor H.R. (2011). Funding models for outreach ophthalmology services. Clin. Exp. Ophthalmol..

[70] Glasson N.M., Crossland L.J., Larkins S.L. (2016). An Innovative Australian Outreach Model of Diabetic Retinopathy Screening in Remote Communities. J. Diabetes. Res..

[71] Glasson N.M., Larkins S.L., Crossland L.J. (2017). What do patients with diabetes and providers think of an innovative Australian model of remote diabetic retinopathy screening? A qualitative study. BMC Health Serv. Res..

[72] Slight C., Marsden J., Raynel S. (2009). The impact of a glaucoma nurse specialist role on glaucoma waiting lists. Nurs. Prax. New Zealand Inc..

[73] O’Connor P.M., Alex Harper C., Brunton C.L., Clews S.J., Haymes S.A., Keeffe J.E. (2012). Shared care for chronic eye diseases: Perspectives of ophthalmologists, optometrists and patients. Med. J. Aust..

[74] Turner A.W., Mulholland W.J., Taylor H.R. (2011). Coordination of outreach eye services in remote Australia. Clin. Exp. Ophthalmol..

[75] Kumar S., Yogesan K., Hudson B., Tay-Kearney M.-L., Constable I. (2006). Emergency eye care in rural Australia: Role of internet. Eye.

[76] Johnson K.A., Meyer J., Yazar S., Turner A.W. (2015). Real-time teleophthalmology in rural Western Australia. Aust. J. Rural. Health.

[77] Hall G., Hennessy M., Barton J., Coroneo M. (2005). Teleophthalmology-assisted corneal foreign body removal in a rural hospital. Telemed. J. E-Health.

[78] McGlacken-Byrne A., Turner A.W., Drinkwater J. (2019). Review of cataract surgery in rural north-Western Australia with the Lions Outback Vision. Clin. Exp. Ophthalmol..

[79] Host B.K., Turner A.W., Muir J. (2018). Real-time teleophthalmology video consultation: An analysis of patient satisfaction in rural Western Australia. Clin. Exp. Optom..

[80] Kumar S., Tay-Kearney M.L., Chaves F., Constable I.J., Yogesan K. (2006). Remote ophthalmology services: Cost comparison of telemedicine and alternative service delivery options. J. Telemed. Telecare.

[81] Razavi H., Copeland S.P., Turner A.W. (2017). Increasing the impact of teleophthalmology in Australia: Analysis of structural and economic drivers in a state service. Aust. J. Rural. Health.

[82] Allen P., Jessup B., Khanal S., Baker-Smith V., Obamiro K., Barnett T. (2021). Distribution and Location Stability of the Australian Ophthalmology Workforce: 2014–2019. Int. J. Environ. Res. Public Health.

[83] Lo T.C., Rogers S.L., Hall A.J., Lim L.L. (2019). Differences in practice of ophthalmology by gender in Australia. Clin. Exp. Ophthalmol..

[84] Danesh-Meyer H.V., Deva N.C., Ku J.Y., Carroll S.C., Tan Y.W., Gamble G. (2007). Differences in practice and personal profiles between male and female ophthalmologists. Clin. Exp. Ophthalmol..

[85] Creed P.A., Searle J., Rogers M.E. (2010). Medical specialty prestige and lifestyle preferences for medical students. Soc. Sci. Med..

[86] McGrail M., O’Sullivan B., Gurney T. (2021). Critically reviewing the policies used by colleges to select doctors for specialty training: A kink in the rural pathway. Aust. J. Rural. Health.

[87] Australian Taxation Office (2019). Taxation Statistics 2018–19.

[88] Optometry Australia Encouraging Optometrists to Identify If They Are Aboriginal and/or Torres Strait Islander at Membership Renewal. https://www.optometry.org.au/national_state_initiatives/encouraging-optometrists-to-identify-if-they-are-aboriginal-and-or-torres-strait-islander-at-membership-renewal/.

[89] Tan I.J., Dobson L.P., Bartnik S., Muir J., Turner A.W. (2017). Real-time teleophthalmology versus face-to-face consultation: A systematic review. J. Telemed. Telecare.

[90] Walsh L., Hong S.C., Chalakkal R.J., Ogbuehi K.C. (2021). A Systematic Review of Current Teleophthalmology Services in New Zealand Compared to the Four Comparable Countries of the United Kingdom, Australia, United States of America (USA) and Canada. Clin. Ophthalmol..

[91] McGrail M.R., O’Sullivan B.G., Russell D.J. (2018). Rural training pathways: The return rate of doctors to work in the same region as their basic medical training. Hum. Resour. Health.

[92] McGrail M.R., O’Sullivan B.G. (2021). Increasing doctors working in specific rural regions through selection from and training in the same region: National evidence from Australia. Hum. Resour. Health.

[93] Puddey I.B., Playford D.E., Mercer A. (2017). Impact of medical student origins on the likelihood of ultimately practicing in areas of low vs. high socio-economic status. BMC Med. Educ..

